# Event-triggered robot self-assessment to aid in autonomy adjustment

**DOI:** 10.3389/frobt.2023.1294533

**Published:** 2024-01-04

**Authors:** Nicholas Conlon, Nisar Ahmed, Daniel Szafir

**Affiliations:** ^1^ Cooperative Human-Robot Intelligence Laboratory, University of Colorado at Boulder, Boulder, CO, United States; ^2^ Interactive Robotics and Novel Technologies Laboratory, University of North Carolina at Chapel Hill, Chapel Hill, NC, United States

**Keywords:** autonomy adjustment, competency assessment, proficiency assessment, human–robot teaming, machine self-confidence, trust, explainable robotics

## Abstract

**Introduction:** Human–robot teams are being called upon to accomplish increasingly complex tasks. During execution, the robot may operate at different levels of autonomy (LOAs), ranging from full robotic autonomy to full human control. For any number of reasons, such as changes in the robot’s surroundings due to the complexities of operating in dynamic and uncertain environments, degradation and damage to the robot platform, or changes in tasking, adjusting the LOA during operations may be necessary to achieve desired mission outcomes. Thus, a critical challenge is understanding when and how the autonomy should be adjusted.

**Methods:** We frame this problem with respect to the robot’s capabilities and limitations, known as robot competency. With this framing, a robot could be granted a level of autonomy in line with its ability to operate with a high degree of competence. First, we propose a Model Quality Assessment metric, which indicates how (un)expected an autonomous robot’s observations are compared to its model predictions. Next, we present an Event-Triggered Generalized Outcome Assessment (ET-GOA) algorithm that uses changes in the Model Quality Assessment above a threshold to selectively execute and report a high-level assessment of the robot’s competency. We validated the Model Quality Assessment metric and the ET-GOA algorithm in both simulated and live robot navigation scenarios.

**Results:** Our experiments found that the Model Quality Assessment was able to respond to unexpected observations. Additionally, our validation of the full ET-GOA algorithm explored how the computational cost and accuracy of the algorithm was impacted across several Model Quality triggering thresholds and with differing amounts of state perturbations.

**Discussion:** Our experimental results combined with a human-in-the-loop demonstration show that Event-Triggered Generalized Outcome Assessment algorithm can facilitate informed autonomy-adjustment decisions based on a robot’s task competency.

## 1 Introduction

Selecting a level of autonomy in line with an autonomous robot’s capabilities is a critical challenge to safe, reliable, and trustworthy robotic deployments. Giving a robot large amounts of autonomy in an environment in which it struggles could lead to damage to the robot and, in the worst case, mission failure. On the other hand, giving a robot less autonomy in an environment where it is quite capable may unnecessarily increase the workload for a human supervisor who must spend more time directly managing or controlling the robot. Robots that can self-assess and report their competency could provide human–robot teams the ability to make informed autonomy-adjustment decisions directly in line with the robots’ abilities.

Consider a scenario where a search-and-rescue (SAR) team is searching for survivors after a disaster. The environment is quite dangerous, so the team decides to employ a semi-autonomous robot to perform a ground search. A human supervisor monitors the robot’s progress from a safe, remote location outside the disaster area. The supervisor receives telemetry data and video from the robot and must use that information to decide whether to take manual control of the robot or allow it to drive autonomously. The supervisor’s decision to trust the robot is based on their perception of the robot’s abilities. However, if there is misalignment between the supervisor’s perception of the robot’s abilities and the robot’s actual competency, the supervisor may inadvertently push the robot beyond its competency boundaries (Hutchins et al., 2015). Unexpected robotic failure can also lead to lower overall trust in the robot, which could lead to the supervisor being less likely to rely on the robot in the future, regardless of the robot’s ability to accomplish the task (Dzindolet et al., 2003; de Visser et al., 2020). To make appropriate autonomy-adjustment decisions, the supervisor needs to understand the robot’s competency and how it may change during the mission.

Recent work has shown that robots that report *a priori* competency self-assessments can align operator perception with the robot’s actual competency, thus improving decision-making and performance in a variable-autonomy navigation task (Conlon et al., 2022b). However, in dynamic and uncertain environments, like the SAR scenario outlined above, an *a priori* confidence assessment can quickly become stale due to environmental changes such as falling debris or unexpected obstacles. In this work, we first develop a metric to monitor *in situ* competency with respect to the robot’s model-based predictions, which we call Model Quality Assessment. We then propose an algorithm called *Event-Triggered Generalized Outcome Assessment* (ET-GOA), which uses the Model Quality Assessment to continually monitor for unexpected state measurements and selectively trigger a high-level self-assessment of the robot’s task objectives. We present evaluation results across several simulated and live robot navigation scenarios, where the environment was unexpected with respect to the robot’s *a priori* knowledge. We next discuss a small demonstration showing how that *in situ* robot competency information can be reported to a human supervisor to facilitate informed autonomy-adjustment decisions. We close with a brief discussion of our results and directions for future work for competency-based autonomy adjustment.

## 2 Background and related work

### 2.1 Variable autonomy

Variable autonomy is a paradigm in robotics where the level of control of a robotic system can change at different points during a task. The level of control can range from a robot with autonomous capabilities acting and making decisions under its own control at one extreme to a human supervisor fully controlling (i.e., teleoperating) and making decisions on behalf of the robot at the other extreme. These levels can be discrete, such as those based on vehicle capabilities found in the autonomous driving literature (Rödel et al., 2014), or can be more fluid and based on the capabilities of the collective human–autonomy team (Methnani et al., 2021). The autonomy level (and adjustments thereof) can also be a function of other factors, such as the distance or data link between the supervisor and robot, the robot’s autonomous competence, or the supervisor’s trust in the robot. Changes to the autonomy level can occur at any time throughout a task or mission. These changes can be initiated by the robot (robot-initiative) (Costen et al., 2022; Mahmud et al., 2023), the human supervisor (human-initiative), or from either the supervisor or the robot (mixed-initiative) (Chiou et al., 2021). While our experiments and demonstration in this manuscript focus on a human-initiative system, robot competency assessment for autonomy adjustment can be applied to mixed-initiative and robot-initiative systems as well.

Autonomy-level changes can be pre-planned and designed into the task (Johnson et al., 2014) or *ad hoc* due to robot degradation or unexpected events (Fong et al., 2002). This work focuses on the latter: to understand when a human supervisor should adjust the autonomy level during the mission due to unplanned events. It is important to note that unplanned events could have a positive or negative impact on the mission, making it more or less difficult for the team to accomplish the mission goals. Previous work in this domain investigated methods for the robot to monitor itself and call for help when necessary (Fong, 2001; Basich et al., 2020; Kuhn et al., 2020) or for the human to take a more active role in monitoring the robot’s abilities and adjust autonomy when necessary (Ramesh et al., 2022; 2021). We take a more collaborative approach that seeks to enable both the robot and the human to monitor metrics of the robot’s task competency to better understand 1) when the robot is more or less capable than previously predicted and 2) when supervisor-initiated autonomy adjustments are necessary. Our approach enables the team to not only understand when the robot is less competent than planned and may need assistance but also realize when the robot is more competent and may be able to operate with increased autonomy.

### 2.2 Robot competency self-assessment

Competency self-assessment enables autonomous agents to assess their capabilities and limitations with respect to task constraints and environmental conditions. This critical information can be used to improve internal decision-making and/or can be communicated to a human partner to improve external decision-making. Pre-mission (*a priori*) self-assessments enable an autonomous agent to assess its competency before the execution of a task or mission. These methods generally compute agent self-confidence based on simulation (Israelsen et al., 2019; Ardón et al., 2020) or previous experience (Frasca et al., 2020). Our recent work showed that reporting of *a priori* self-assessments lead to better choices of reliance (Conlon et al., 2022c) and improvements in performance and trust (Conlon et al., 2022b). However, in dynamic environments, *a priori* assessment is a poor predictor of the agent’s confidence due to factors that are not accounted for before execution, such as environmental changes, task changes, or interactions with other agents. Running *a priori* methods online (periodically) could conceivably capture dynamic competency changes. However, such assessments can waste computational resources if competency has, in fact, not changed or may be too expensive for certain kinds of decision-making agents (Acharya et al., 2022; Conlon et al., 2022a; Gautam et al., 2022).

In mission (*in situ*) self-assessment enables an autonomous agent to assess (or reassess) its competency during task execution. Popular methods such as online behavior classification can identify poor behavior and trigger the agent to halt the operation and ask for help in the event of a failure (Fox et al., 2006; Rojas et al., 2017; Wu et al., 2017). These methods, although able to capture dynamic competency changes, require examples of both good (competent) and poor (incompetent) behaviors, which may be difficult or impossible to acquire in many real-world applications. Another method of *in situ* self-assessment involves monitoring features of the agent’s current state. For example, Gautam et al. (2022) developed a method to monitor deviations from design assumptions, while Ramesh et al. (2022) used the “vitals” of a robot to monitor its health during task execution. Both methods provide a valuable instantaneous snapshot of the agent’s state at a given time, which can indicate performance degradation online; however, neither predicts higher-level task competency (for example, does the degradation actually impact the task outcome?).

In contrast, we propose an algorithm that enables the assessment and communication of high-level task outcome competency both *a priori* and *in situ*. We leverage the method of Generalized Outcome Assessment, which was originally developed as an *a priori* method due to computational cost, to assess a robot’s task outcome competency. We then develop a method of *in situ* Model Quality Assessment that monitors the alignment between the robot’s model predictions and state observations to intelligently choose when the robot should (re-)assess and (re-)communicate task outcome competency. We argue that understanding when and how the robot’s competency has changed will help human supervisors make improved autonomy-adjustment decisions.

## 3 Algorithm development

### 3.1 Modeling the world

We take a model-based approach to competency self-assessment. We define a world model, *M*, as a stochastic model of the robot, its dynamics, and its environment from which trajectories can be sampled. *M* could take the form of a Monte Carlo-based planner (Israelsen, 2019), a black box neural network (Ha and Schmidhuber, 2018; Conlon et al., 2022a), or a high-fidelity simulation environment (Koenig and Howard, 2004; Michel, 2004). Similar modeling paradigms have been referred to as model-based in the reinforcement learning literature (Acharya et al., 2023; Moerland et al., 2023) and digital twins in the simulation literature (Girletti et al., 2020; Phanden et al., 2021; Xu et al., 2021). Within the framework of Factorized Machine Self-Confidence (FaMSeC), the purpose of *M* is to enable the robot to simulate itself, executing the task in a representative environment. Sampling from *M* results in a predicted distribution of trajectories through the robot’s state space, which can be analyzed by our FaMSeC assessments to understand how capable the robot is expected to be.

### 3.2 Factorized Machine Self-Confidence

**FIGURE 1:**
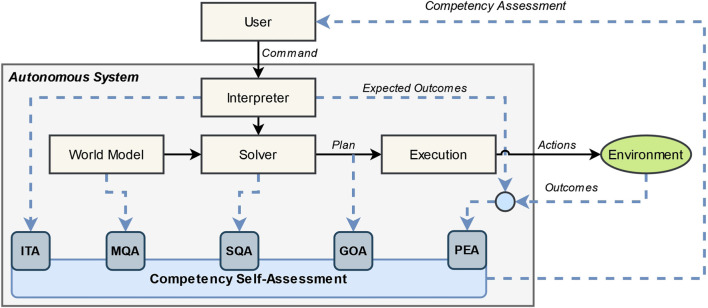
Factorized Machine Self-Confidence framework adapted from Israelsen (2019). Planning and execution components (tan boxes, black arrows) are assessed across five factors (blue rounded boxes, blue dashed lines) to assess the robot’s competency.

To help facilitate informed autonomy-adjustment decisions, we capture changes in robot competency with FaMSeC. FaMSeC is a computational framework that enables an autonomous robot to self-assess across five different dimensions that are theorized to impact competency. A diagram of the FaMSeC framework adapted from Israelsen (2019) is given in Figure 1.

FaMSeC assumes a planning and execution flow commonly found in autonomous systems, which is shown as tan boxes with black lines in Figure 1. First, the user issues a command (or a task) to the robot. Next, the robot must interpret or translate that task. The interpreted task, along with the robot’s world model, is then used by the robot’s solver to generate a plan, and that plan is then executed through interactions with the environment.

The assessment mechanism—shown as rounded blue boxes connected with blue dashed lines—evaluates each planning and execution component to varying degrees, which captures the robot’s overall competency. *Interpretation of Task Assessment (ITA)* assesses how well the agent has interpreted the user commands. *Model Quality Assessment (MQA)* assesses how well the agent’s world model aligns with reality. *Solver Quality Assessment (SQA)* assesses how well the agent’s solvers and policies align with optimal or trusted solvers and policies. *Generalized Outcome Assessment (GOA)* assesses the plan to predict the degree to which the agent will achieve user-defined outcomes. *Past Experience Assessment (PEA)* assesses how the robot has performed in previous and similar problem instances by analyzing expected and actual outcomes. A combination of some or all factors can be reported to a human user to calibrate their mental model to the robot’s predicted competency, with respect to a given task and environment. For a thorough treatment on Factorized Machine Self-Confidence, please refer to Israelsen (2019).

To date, only the GOA and Solver Quality Assessment (SQA) metrics have been fully implemented and validated. Outcome Assessment is a powerful tool to calibrate users to robot capabilities but only as an *a priori* metric. Toward *in situ* competency assessment, this work first proposes a novel MQA FaMSeC metric. We then develop an algorithm that combines MQA with an existing GOA to enable fast online monitoring and selective assessment of task outcome competency.

### 3.3 Assessing task outcome competency

To assess task outcome competency, we leverage Generalized Outcome Assessment (*GOA*) (Conlon et al., 2022a), an extension of the original Outcome Assessment proposed by Israelsen (2019). For brevity, we refer to this as Outcome Assessment or *GOA* henceforth. *GOA* begins by simulating task execution by sampling state predictions from a world model-based distribution *π*(*s*_*t*+1_|*s*_*t*_, *a*_*t*_), which results in a distribution of predictions for a target outcome of interest. Examples of target outcomes include goals accomplished or task completion time.

The outcome predictions are then ranked according to their desirability to the user such that for outcomes *z*_*i*_ and *z*_*j*_, *z*_*i*_ < *z*_*j*_ if *z*_*i*_ is less desirable than *z*_*j*_, and all outcomes less than a threshold z_*_ are considered undesirable. For example, a user might desire a task completion time of no more than z_*_ = 60 s, where a prediction of a late completion at *z*_*i*_ = 65 s is less desirable than a prediction of an early completion at *z*_*j*_ = 45 s. Next, *GOA* analyzes the ranked predictions according to the ratio of the upper partial moment to lower partial moment (Wojt, 2009):

$$\frac{\text{UPM}}{\text{LPM}}{|}_{z_{*}}=\frac{\sum_{z_{j}\geq z_{*}}\left(z_{j}-z_{*}+1\right)*P\left(z_{j}\right)}{\sum_{z_{m}\leq z_{*}}\left(z_{*}-z_{m}\right)*P\left(z_{m}\right)}.$$

The $\frac{UPM}{LPM}$ statistic weights the probability of an outcome by its distance from z_*_. Because it ranges from negative to positive infinity, it is transformed to the range [0, 1] through a standard sigmoid function:

$$GOA=\frac{1}{1+e^{-\frac{\text{UPM}}{\text{LPM}}{|}_{z_{*}}}}.$$

The value of *GOA* is an indicator of the robot’s confidence in achieving outcomes equal to or more desirable than z_*_. We expect that if the robot’s world model predictions are generally above z_*_, then, *GOA* tends toward 1 (higher confidence), and conversely, if the world model predictions are generally below z_*_, we expect *GOA* to tend toward 0 (lower confidence). The value of *GOA* can be reported as a raw numeric ∈ [0, 1] or mapped to a semantic label indicating confidence. For our quantitative experiments covered later in this manuscript, we analyzed raw numerical *GOA* confidence values. For our qualitative human-in-the-loop demonstration, we reported the semantic labels using the mappings *very bad confidence* (*GOA* ∈ [0, 0.25)), *bad confidence* (*GOA* ∈ [0.25, 0.4)), *fair confidence* (*GOA* ∈ [0.4, 0.6)), *good confidence* (*GOA* ∈ [6, 0.75]), and *very good confidence* (*GOA* ∈ [0.75, 1.0)).

It is important to note that *GOA* can be an expensive operation to run online due to sampling of potentially complex world models. To understand how task outcome competency has changed *in situ*, a designer must cope with a trade-off between an accurate understanding of task outcome competency and the computational expenditures needed to assess the said task outcome competency. One way to address this trade-off is to provide the robot the ability to intelligently choose to self-assess based on predicting when its competency has potentially changed.

### 3.4 Developing the Model Quality Assessment

To estimate when competency may have changed, we implemented a new metric for Factorized Machine Self-Confidence called MQA. The MQA was defined by Israelsen (2019) as assessing “how well measurements and events predicted by an autonomous system model align with what it actually should observe in reality.” We argue that MQA should be thought of as a distance function between an autonomous agent’s model prediction and real observations or measurements gained through interacting with the environment. Furthermore, we believe that MQA should be bounded within a small range to align with the other FaMSeC factors. In order to assess based on live measurements, model quality should also be fast enough to run online. We propose a general form of Model Quality Assessment as follows:

$$MQA=f\left(y,\hat{y}\right)\in \left[0,1\right],$$

where MQA should tend toward 1 if there is high alignment between the model predictions $\hat{y}$ and real measurements *y* and tend toward 0 otherwise.

One promising method that fits our requirements is the surprise index (*SI*). *SI* is defined as the sum of probabilities of more extreme (or less probable) events than an observed event given a probabilistic model (Zagorecki et al., 2015). For a given event, *e*, modeled by probability density function *π*, *SI* is computed by summing over the probabilities of more extreme events in *π*:

$$SI\left(\pi \left(e\right),\pi \right)=\int_{\pi \left(i\right)<\pi \left(e\right)}\pi \left(i\right)d\pi .$$

The surprise index can be thought of as how (in)compatible an observation *e* is given a set of possible events predicted by *π*. This is similar to the more well-known entropy-based surprise (Benish, 1999; Baldi and Itti, 2010). However, whereas entropy-based surprise is unbounded, the surprise index is bounded between zero (most surprising) and one (least surprising). *SI* also shares similarities with the tail probability or the *p*-value, given the hypothesis that *e* is from the distribution *π*. A large *p*-value (*SI* tending toward one) indicates that *e* may have been sampled from *π*, while a small *p*-value (*SI* tending toward zero) indicates strong evidence to the contrary.

For an autonomous agent with multivariate state *s*_*t*_ ∈ *S* at time *t*, we define the Model Quality Assessment at time *t* as the minimum of the surprise index across the state marginals, given a state observation *s*_*t*_ and state prediction *π*_*t*_. Here, *π*_*t*_ is predicted by the agent’s world model, and *s*_*t*_ is the state observation received at time *t*. We marginalize the state and compute the metric over marginals included in indicator list *I*. *I* is a list of designer-defined state elements that should be monitored to assess competency; for example, the robot’s (*x*, *y*, *z*) position may be included in *I*, while its current control state “teleoperation” or “autonomous” may not. This gives us a succinct surprise index-based MQA formulation using only essential state elements:

$$MQA_{t}=\underset{i\in I}{\min}SI\left(s_{t,i},\pi_{t,i}\right)\in \left[0,1\right].$$

Continuous monitoring of the MQA during task execution can provide information about how competent the agent is in a given environment and how that may change *in situ*. Moreover, we can use the instantaneous value of the MQA as a trigger for the agent to re-assess the higher-level outcome of the task. In other words, a waning MQA indicates that the agent’s world model predictions have diverged from measurements and can be an indicator that the agent’s higher-level task competency has changed. In this work, we say that the agent should reassess higher-level task outcomes if the MQA falls below a designer-defined threshold *δ*. Additionally, it is important to note that MQA and GOA operate over world model predictions of the same form, (*s*_*t*_, *a*_*t*_, *s*_*t*+1_). Trajectories sampled by GOA to assess outcomes can be used by MQA, along with real observations, to assess model quality.

### 3.5 Putting it together: the Event-Triggered Generalized Outcome Assessment algorithm

We combine the Model Quality Assessment and the Generalized Outcome Assessment into an algorithm for *in situ* competency self-assessment called ET-GOA. The algorithm is presented in Algorithm 1 and can be broken up into two components: (1) *a priori* (or before task execution) and (2) *in situ* (or during task execution).

*Before task execution (lines 1–5)*: Line 1 takes as input a world model *M*, a task specification *T*, a set of GOA thresholds *Z*, an MQA threshold *δ*, and a set of MQA indicators *I*. Next (line 2), *M* is used to simulate the execution of task *T*, given an initial state *s*_0_. This results in a set of predictions ${\left[\pi_{t}\right]}_{t=0:N}$ for each time step *t*. The predictions for each time step are stored in an experience buffer (line 3) and then used to compute the initial Generalized Outcome Assessment (line 4), which can be reported to an operator (line 5).

Algorithm 1 Event-Triggered Generalized Outcome Assessment.

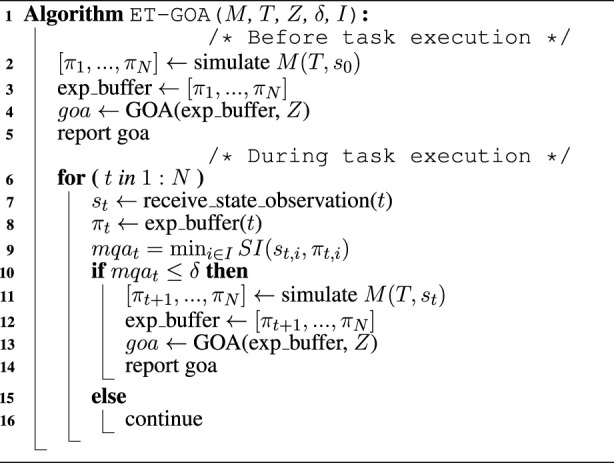

*During task execution (lines 6–16)*: The agent receives state observation *s*_*t*_ at time *t* (line 7). It then retrieves the state predictions *π*_*t*_ from the experience buffer (line 8). Next, the algorithm computes the MQA (line 9). If *mqa*_*t*_ is below the threshold *δ*, an anomalous or unexpected state observation has been received, and task outcome confidence should be reassessed (line 10). In this case, a new set of predictions *π*_*t*+1:*N*_ is sampled from simulating *M* (line 11) and saved in the experience buffer (line 12). A new GOA is then computed using the newly updated experience buffer (line 13), and the associated competency is reported to the operator (line 14). If, on the other hand, *mqa*_*t*_ is above the threshold *δ*, this indicates that the agent’s predictions align with its observations, and no confidence update is needed at this time (line 16). This loop (line 6) continues for the duration of the task, comparing state prediction *π*_*t*_ to the state observation *s*_*t*_ and (if necessary) computing and reporting updates to the robot’s task competency.

**FIGURE 2:**
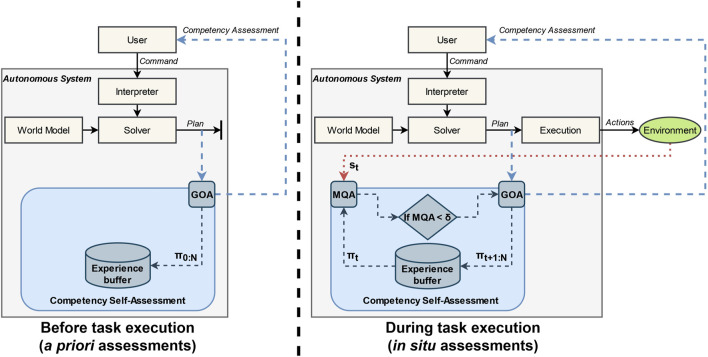
System diagram for the Event-Triggered Generalized Outcome Assessment algorithm. The left side shows the *a priori* or before-task behavior showing the initial outcome assessment (GOA), competency reporting, and storing of world model predictions. The right side shows the *in situ* or during-task behavior showing the Model Quality Assessment at each time step, and, if triggered, *in situ* outcome assessments, competency reporting, and updating of world model predictions.

The flowchart of the Generalized Outcome Assessment algorithm is shown in Figure 2. The left side of the figure shows the *a priori* portion of the algorithm: given a user command or task, the system generates an initial plan, which is assessed by Generalized Outcome Assessment, and then communicated to a user to improve *a priori* decision-making. The world model state predictions for each time step, *π*_0:*N*_, generated by GOA, are also saved in the experience buffer. The right side of the figure shows the *in situ* portion of the algorithm: at time *t*, the state measurement from the environment, *s*_*t*_, and world model prediction, *π*_*t*_, are used to compute the MQA. If the assessment is less than threshold *δ*, then GOA is executed and reported to the user for improved *in situ* situational awareness and decision-making. After GOA is run, the experience buffer is updated with new world model predictions *π*_*t*+1:*N*_. If the assessment is greater than the threshold, then no reassessment is needed. The loop of continuous Model Quality Assessment and selective Generalized Outcome Assessment continues for the duration of the task.

## 4 Experimental design

To validate the Model Quality Assessment and the Event-Triggered Generalized Outcome Assessment algorithm, we designed and executed experiments across both simulated and live scenarios.

### 4.1 Research questions

We developed four core research questions to analyze Model Quality Assessment and the Event-Triggered Generalized Outcome Assessment algorithm:

1. *How does the Model Quality Assessment respond to different types of perturbations?* We hypothesize that the MQA will be lower for unexpected state measurements and higher for expected state measurements. This would indicate that MQA can capture misalignment between the robot’s world model and reality.
2. *How does the triggering threshold impact the accuracy of ET-GOA?* We hypothesize that GOA accuracy will increase proportionally to the *δ* threshold. We believe that a higher *δ* threshold should increase the sensitivity of MQA and increase the number of GOA triggers.
3. *How does the triggering threshold impact the computational complexity of ET-GOA?* We hypothesize that the computational complexity of the ET-GOA algorithm will increase proportionally to the *δ* threshold. The increase in threshold will increase the number of GOA triggers, which will result in higher computational complexity.
4. *Does ET-GOA perform similarly in simulation and on a live platform?* We hypothesize that the ET-GOA algorithm on a live platform will respond to state perturbations similar to the simulated robot. We expect to see the same general trends in MQA behavior between our simulated and live experiments.

### 4.2 Robot state, planning, and competency self-assessment

For both the simulated and live experiments, the robot’s state took the form (*x*,*y*,*z*)_*t*_, where (*x*, *y*, *z*) is the position in meters in a global frame and *t* is the time in seconds. To generate waypoints, we used a rapidly exploring random tree (*RRT*) planner, which is a common stochastic method for generating motion plans in an obstacle-rich environment (Lavalle, 1998). We used a simple proportional derivative (PD) controller to compute velocity actions between individual waypoints. To avoid confounding our experiments, we did not include any autonomous replanning or obstacle avoidance maneuvers. In other words, our robot had only limited competency while driving autonomously. If any predetermined path was blocked, the robot was programmed to automatically stop to prevent physical collision with obstacles.

The robot’s world model was an instance of the Webots high-fidelity simulator (Michel, 2004). The simulator was programmed with a copy of the robot as well as copies of all obstacles the robot had knowledge of at the current time step. If the robot sensed an obstacle in the execution environment using its front-facing camera, the world model was updated with a simulated obstacle of similar size and position. When the ET-GOA algorithm was active, the robot had the ability to query the world model on demand for self-assessments.

Generalized Outcome Assessment was computed using 10 Monte Carlo rollouts of the world model robot navigating from the “real” robot’s current location along the waypoints, given all known obstacles. Our experiments focused on a single outcome of interest for GOA: *autonomous driving time*. This was the time the robot spent in an autonomous driving state. To facilitate a more detailed analysis of our ET-GOA algorithm, we separated the time spent running a GOA assessment from the time spent autonomously driving in the environment. Thus, the autonomous driving time outcome was equal to the time spent driving minus the time spent running GOA. We specified a maximum desired autonomous driving time of 60 s. This meant that GOA was parameterized by z_*_ = 60, and the assessment returned the robot’s confidence in successfully navigating to the goal within 60 s of the cumulative time spent in the autonomous driving state. For our experiments, the raw GOA value in [0,1] was used, and for the demonstration, we mapped the raw GOA value to a semantic label, as in Section 3.3. Note that we could have instead looked at other outcomes of interest such as minimum or maximum velocity, obstacle hit, or exceedances of operational thresholds. The world model predictions generated during GOA were saved in an experience buffer for use by MQA.

Model Quality Assessment was run each second using the current world model predictions in the experience buffer and state indicators *I* = [*x*, *y*, *z*]. The choice of indicator set is of critical importance and should include state elements that the designer foresees as being impacted by changes in competency. The world model predictions at each time step for each indicator *i* ∈ *I* were modeled as a normal distribution, $N\left(\mu_{i},\sigma_{i}\right)$ . For a real observation *x*_*i*_, the surprise index was then the sum of the lower- and upper-tail CDF, *F*(*x*|*x* < *o*_*i*_ ∪ *x* > *o*_*i*_). In other words, the sum of probabilities of all observations more extreme than *o*_*i*_. If any *in situ* re-assessments occurred, the experience buffer was flushed and updated with the latest world model predictions.

### 4.3 Simulation experiment overview

Our simulation experiments were run on a custom-built Windows 10 PC with an Intel Core i7 3.4 GHz CPU, 32 GB RAM, and NVIDIA RTX 3060 GPU. We used the Webots simulator customized for our specific use case. The simulation environment was a 4 × 10 m space with a single ground robot of approximate size, shape, and capability as a Clearpath Jackal. The robot was equipped with a notional sensor capable of sensing obstacles within a 2 m radius from the robot. The robot also received accurate position information at all times from the simulation. The robot’s physical capabilities were limited to basic waypoint following and emergency stopping, and deviation from the planned waypoints would require human control. In other words, our robot was only moderately competent.

To investigate our first research question, we tasked the robot with driving from point A to point B along a fixed set of waypoints. We varied the amount and type of state measurement perturbation the robot experienced as well as how well those perturbations were captured in the robot’s world model. We developed the following three conditions:

1. *Accurate world model*: The actual execution environment contained an area of high transition noise, which we programmed to 1) reduce the robot’s intended speed by 50% and 2) added random Gaussian noise $\left(\mu =0,\sigma =0.5\frac{m}{s}\right)$ to the robot’s velocity actions. The robot’s world model was provided accurate *a priori* information about all obstacles in the environment. This condition represented the baseline case where the robot’s world model accurately captured all information about the environment.
2. *Unexpected transition noise*: The actual execution environment contained an area of high transition noise, which we programmed to (1) reduce the robot’s intended speed by 50% and (2) added random Gaussian noise $\left(\mu =0,\sigma =0.5\frac{m}{s}\right)$ to the robot’s velocity actions. Neither could the robot directly sense the area nor did the robot’s world model contain any *a priori* information about this area. This condition represented a case where the robot experienced increasingly unexpected position measurements *in situ* due to the impact of the area.
3. *Unexpected blocked path*: The actual execution environment contained a wall blocking the robot’s ability to navigate to the goal. The robot’s world model had no *a priori* information of this wall. This condition represented a case where the robot experienced both an instantaneous unexpected obstacle measurement when it sensed the wall and increasingly unexpected position measurements as it was unable to continue the navigation due to the wall.

**FIGURE 3:**
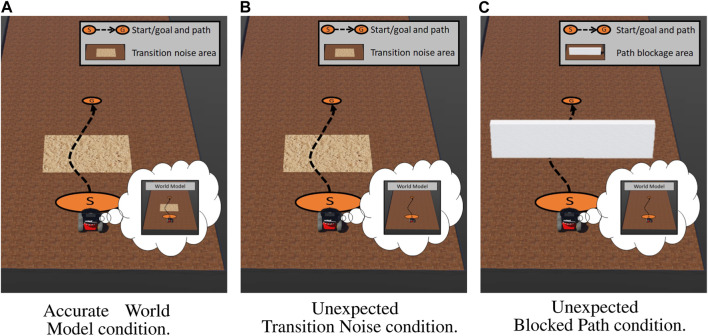
Simulation study environment configurations with the approximate path shown in a black dotted line from start (S) to the goal (G). **(A)** The robot’s world model had accurate knowledge about an area of high transition noise. **(B)** The robot’s world model did not have accurate knowledge about an area of high transition noise. **(C)** The robot’s world model did not have knowledge about a wall blocking the path to the goal. The contents of the robot’s world model can be seen in each robot’s thought bubble. **(A)** Accurate world model condition. **(B)** Unexpected transition noise condition. **(C)** Unexpected blocked path condition.

All three conditions are shown in Figure 3. It should be noted that the only difference between the accurate world model and unexpected transition noise conditions was the *a priori* knowledge provided to the world model.

To investigate our second and third research questions, we tasked the robot with driving from start (S) to goal (G) along a fixed set of waypoints. The execution environment was a combination of the transition noise and blocked path conditions. Here, the robot’s path first took it over the high-transition-noise area, followed by the blocked path area *en route* to the goal. The robot’s world model had no *a priori* knowledge about the obstacles. We endowed the robot with a sensor capable of sensing the transition noise area and the wall. As the robot navigated through the environment, it added previously unknown obstacles to its world model as they were sensed. GOA was computed using 10 Monte Carlo runs of the robot’s world model, which was updated with all *a priori* information and *in situ* observations. For MQA, we restricted the world model’s minimum state variance to 1 m to prevent the ET-GOA algorithm from sampling a degenerate (or constant) state distribution in the event the world model was overly confident. Our conditions consisted of seven ET-GOA triggering thresholds, *δ* = (0.0, 0.1, 0.25, 0.5, 0.75, 0.9, 1.0).

### 4.4 Live experiment overview

Our live experiments were conducted in the University of Colorado Boulder Smead Aerospace Engineering Sciences Autonomous Systems Programming, Evaluation, and Networking (ASPEN) Laboratory. We used a Clearpath Jackal equipped with an onboard computer, wireless communication, and front-facing camera for basic object detection. We affixed AR tags on the obstacles, which gave the robot accurate measurements of the obstacle type and reduced any confounds relating to the accuracy of detection algorithms. The ET-GOA algorithm as well as all task planning was conducted off board on an Ubuntu 20.04 laptop with an Intel Core i7 2.3 GHz CPU, 16 GB RAM, and NVIDIA RTX A3000 GPU. All communication between the robot, camera, and laptop was conducted over the Robot Operating System (ROS), a popular robotics middleware (Quigley et al., 2009). The robot’s mission area was a 4 × 10 m area within the ASPEN Laboratory equipped with a VICON camera system, which was used for accurate position estimates. Similar to the simulated robot, the live robot’s physical capabilities were limited to basic waypoint following and emergency stopping, and deviation from the planned waypoints would require human control.

Our live evaluation of MQA mirrored the simulation conditions covered in Section 4.3. Our first two conditions evaluated expected and unexpected transition noise. Instead of simulated transition noise, the real environment contained a set of uneven sandbags along the robot’s prescribed path, which created transition disturbances as the robot drove over them. Under the *accurate world model* condition, we provided the robot’s world model accurate *a priori* information about the sandbag location and impact to velocity. Under the *unexpected transition noise* condition, we did not provide the robot any *a priori* information about the sandbags. Our third condition, *unexpected blocked path*, evaluated the MQA response when the robot could not continue on its prescribed path. Instead of a simulated wall blocking the path, the real environment contained a large cardboard box blocking the prescribed path. An ARTag was affixed to the box, which helped the robot identify the obstacles and update its world model accordingly.

**FIGURE 4:**
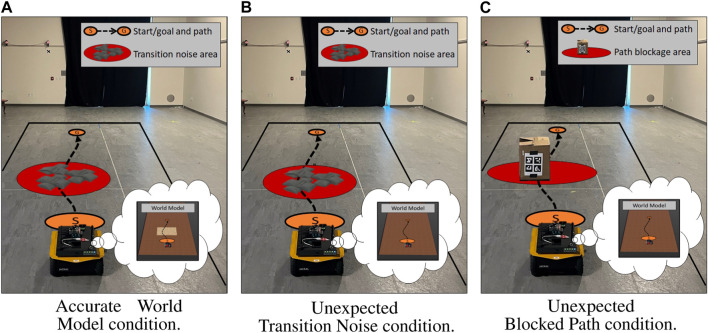
Live study environment configurations with the approximate path shown in a black dotted line from start (S) to the goal (G). **(A)** The baseline condition contained a set of sandbags that the robot’s world model was given full knowledge about. **(B)** The area contained a set of sandbags that the robot’s world model did not have knowledge of. **(C)** A popup obstacle was placed in front of the robot shortly after the task began, which the robot’s world model had no *a priori* knowledge about. The contents of the world model can be observed in each robot’s thought bubble. **(A)** Accurate world model condition. **(B)** Unexpected transition noise condition. **(C)** Unexpected blocked path condition.

For each episode, the robot attempted to drive approximately 4 m, during which it experienced the condition. All three conditions are shown in Figure 4. Similar to our simulation experiments, the accurate world model condition was identical to the unexpected transition noise scenario, except that the world model had full knowledge about the sandbags.

### 4.5 Measures

We used three primary measures to evaluate MQA and the ET-GOA algorithm:

1. *Model quality response*: The goal of this measure was to understand how well MQA captured state measurement perturbations at various times throughout the task execution and with varying amounts of alignment between the world model and reality. We measured the raw MQA value at each time step and computed the mean across the task.
2. *Computational cost*: The goal of this measure was to understand the computational efficiency of ET-GOA. We measured the time in seconds that the robot spent executing a self-assessment (Model Quality Assessment and Generalized Outcome Assessment) during the duration of the task.
3. *Outcome assessment accuracy*: The goal of this measure was to understand how accurate the ET-GOA-triggered Generalized Outcome Assessments were. We computed the mean squared error (MSE) between the ET-GOA-triggered Generalized Outcome Assessments and a ground truth periodic GOA at each time step. For time steps where ET-GOA did not trigger an updated Outcome Assessment, we reused the last ET-GOA-computed Outcome Assessment in the MSE computation.

We utilized statistical significance testing in our analysis of Research Question 1. For analyzing a main effect across conditions, we performed a one-way analysis of variance (ANOVA), measuring the effect size by partial eta-squared $\left(\eta_{p}^{2}\right)$ . To analyze differences between individual conditions, we utilized Tukey’s honestly significant difference (HSD) test with Cohen’s *d* effect size measure. For all statistical testing, we set *α* = 0.05. For research questions 2 and 3, we analyze the correlation across thresholds using Pearson’s correlation. For research question 4, we provide a higher-level analysis and discussion of our results.

## 5 Results

### 5.1 Model Quality Assessment response in simulated scenarios

**FIGURE 5:**
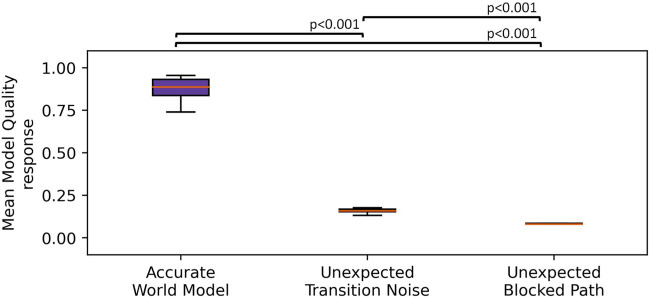
Boxplot showing the MQA response across three simulated conditions. The whiskers indicate the first quartile ±1.5× interquartile range. We observed that the unexpected transition noise and unexpected blocked path conditions showed significantly lower MQA than where the robot was given an accurate world model.

We executed 20 episodes per condition and measured the MQA response at each time step and then computed the mean MQA across each task. A one-way ANOVA indicated a significant main effect across the three conditions, $F\left(2,57\right)=2464.6,p<0.001,\eta_{p}^{2}=0.99$ . Further analysis using Tukey’s HSD test showed a significant decrease in MQA between the accurate world model condition (*M* = 0.88) and the unexpected transition noise condition (*M* = 0.16), *p* < 0.001, *d* = 18.2, a significant decrease in MQA between the accurate world model condition and the unexpected blocked path condition (*M* = 0.08), *p* < 0.001, *d* = 20.1, and a significant decrease in MQA between the unexpected transition noise condition and the unexpected blocked path condition, *p* < 0.001, *d* = 1.9. This supported our hypothesis that MQA could capture unexpected perturbations to the robot’s state. Additionally, these results showed that different types of perturbations elicited different MQA responses. A plot of the mean MQA response across the three simulated conditions is given in Figure 5.

### 5.2 ET-GOA response in simulated scenarios

**FIGURE 6:**
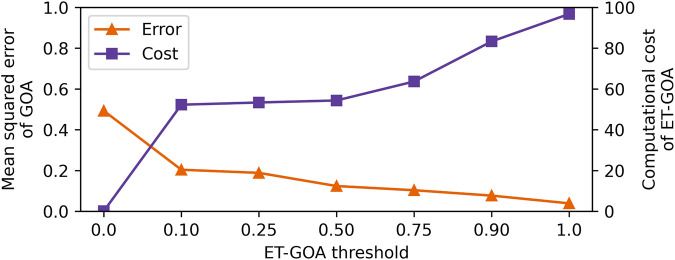
Plot showing ET-GOA evaluated across seven triggering thresholds. The orange triangles indicate the mean squared error, which decreases with an increasing threshold. The purple squares indicate the percentage of task time spent running the ET-GOA algorithm, which increases with an increasing threshold. There is a trade-off between ET-GOA self-assessment accuracy and the computational cost of executing said self-assessments.

To understand the accuracy of the ET-GOA algorithm, we computed the MSE between the ET-GOA-triggered confidence predictions and a ground truth GOA at each time step. Note lower MSE equates to more accurate predictions. The results of Pearson’s correlation indicated that there was a significant negative correlation between the *δ* threshold and GOA error, *r*(138) = −0.75, *p* < 0.001, *R* ^2^ = 0.57. This can be seen as the orange triangles in Figure 6. The lower thresholds rarely triggered GOA, while higher thresholds captured *in situ* competency changes due to triggering GOA more often. The higher the rate of triggered GOA, the more in line with the ground truth the robot’s *in situ* performance predictions were. These findings supported our second hypothesis that GOA accuracy would increase (error would decrease) proportionally to the triggering threshold.

We measured the computational cost as the percentage of task time in seconds that the robot spent running the ET-GOA algorithm in the periodic MQA assessment and the triggered GOA portions. We found a significant positive correlation between the *δ* threshold and computational cost, *r*(138) = 0.86, *p* < 0.001, *R* ^2^ = 0.74. This can be seen as the purple squares in Figure 6. Lower thresholds led to less computational cost, and higher thresholds led to higher computational costs. At the extremes, a threshold of *δ* = 0 leads to exactly one *a priori* self-assessment because the sensitivity of the trigger is at a minimum, and a threshold of *δ* = 1 induced behavior similar to a periodic assessment because the sensitivity of the trigger was at its maximum. Further analysis revealed that the per-observation MQA was quite fast (M = 0.003 s), while the triggered GOA was the computational bottleneck (M = 46.17 s) of the algorithm. This is not unexpected as the GOA algorithm executed several Monte Carlo rollouts of a high-fidelity simulator each time it was triggered by MQA. Additionally, the goal of MQA is to limit the computational expenditure of robot competency assessment by triggering outcome assessments only when necessary. These findings supported our third hypothesis that the computational cost of ET-GOA increased proportionally to the triggering threshold.

**FIGURE 7:**
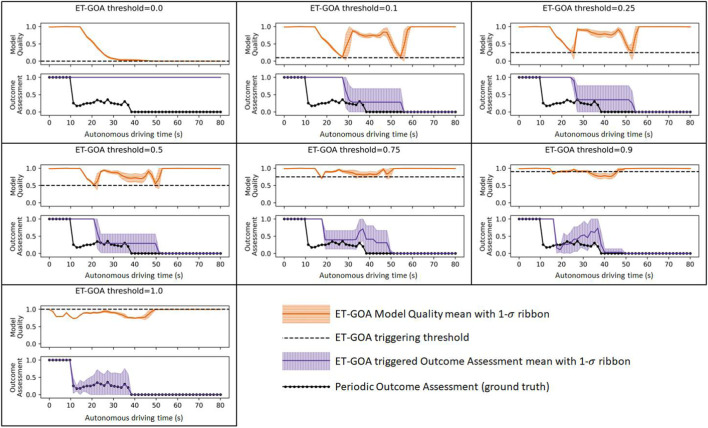
MQA and GOA measured across several thresholds during task executions. The black dashed line indicates the MQA threshold for triggering GOA.

Figure 7 shows all of our simulation data in a single plot. Each subplot shows the data for each threshold we examined. For a given threshold, we plotted the MQA and GOA over autonomous driving time, along with the 1*σ* error bounds. Recall that autonomous driving time ignores the time the robot spends running GOA and allows for a straightforward comparison of the ground truth and ET-GOA-triggered assessments at each step. The *δ* threshold for the event triggering is shown as a red dotted line on the Model Quality Assessment plot. The periodic GOA (ground truth) is shown as a series of black circles on the Outcome Assessment plot. The ground truth indicates that the robot should initially be quite confident in task success. However, at approximately time *t* = 20 s, when the robot encounters (and learns of) the high-transition-noise area, its confidence should decrease because Monte Carlo simulations of that area result in lower probability of success. At approximately *t* = 40 s, the robot encounters a previously unknown wall. With knowledge of the wall blocking the path, the robot’s confidence is now essentially zero. Moving left to right and top to bottom, we can see that the number of triggers increases as the threshold increases. The increase in triggers causes an increase in *in situ* GOA assessments. The higher frequency of assessments leads to a more accurate overall picture of task outcome confidence but at a cost of increased computation time, as we discussed earlier. The increase in accuracy can be seen in the plots as the triggered GOA tightly enveloping the periodic ground truth GOA.

Our simulation experiments helped us answer our first three research questions. First, we found evidence supporting our hypothesis that MQA could capture unexpected state perturbations across three scenarios, where the world model had varying knowledge of the environment. Second, we found evidence supporting our hypothesis that GOA accuracy would increase proportionally to the triggering threshold. Third, we found evidence supporting out hypothesis that computational cost would increase proportionally to the triggering threshold. In investigating the second and third hypotheses, we found that there is a distinct trade-off designers must make with respect to computational cost and GOA prediction accuracy. Additionally, both the MQA and the ET-GOA algorithm showed the behavior we were expecting within the experiments.

### 5.3 Model quality response in live scenarios

**FIGURE 8:**
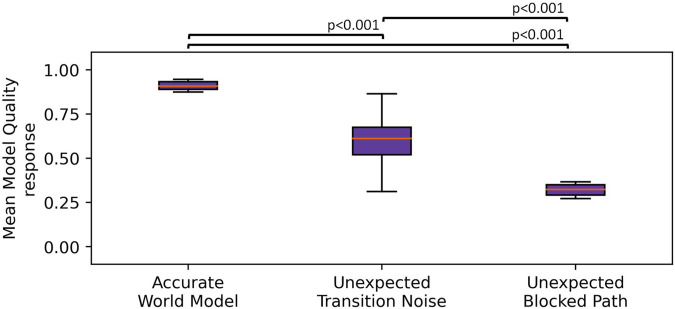
Boxplot showing the MQA response across the three live conditions. The whiskers indicate the first ±1.5× interquartile range. We observed that the unexpected transition noise and unexpected blocked path conditions showed significantly lower MQA than when the robot was given an accurate world model.

We executed 20 episodes per condition and measured the MQA response at each timestamp and then computed the mean MQA across each task. We found a significant effect of the condition on MQA, $F\left(2,57\right)=232.7,p<0.001,\eta_{p}^{2}=0.89$ . Further analysis using Tukey’s HSD test showed a significant decrease in MQA between the accurate world model condition (*M* = 0.91) and the unexpected transition noise condition (*M* = 0.60), *p* < 0.001, *d* = 3.3, a significant decrease in MQA between the accurate world model condition and the unexpected blocked path condition (*M* = 0.26) *p* < 0.001, *d* = 6.8, and a significant decrease in MQA between the unexpected transition noise condition and the unexpected blocked path condition, *p* < 0.001, *d* = 3.5. This supports our hypothesis that the MQA response on a live robot captured unexpected perturbations to its state. We observed behavior similar to that of our simulation experiments, albeit with much smaller effect sizes. These smaller effect sizes are most likely due to the mean MQA being slightly closer across the live scenario conditions than across the simulated conditions. A plot of the mean MQA response across the three live conditions is given in Figure 8.

Our live experiments helped us answer our research question 4. We found evidence to support our hypothesis that MQA would show a similar response between the simulation and live studies. We observed that MQA did, in fact, perform in line with our expectations across the three scenarios. This reinforced our belief that MQA and the ET-GOA algorithm could be used on live robot platforms to calibrate a human supervisor’s understanding of robot competency and facilitate supervisor-initiated autonomy-adjustment decisions.

### 5.4 A demonstration and discussion of ET-GOA for autonomy adjustment

Our experiments showed that 1) the Model Quality Assessment can capture events that were unexpected with respect to the robot’s world model and 2) that the ET-GOA algorithm can respond to changes in Model Quality Assessment with updated task outcome assessments. However, to understand how ET-GOA may add value to human–robot teaming, we must evaluate with a human in the loop. To that end, we developed a proof-of-concept scenario where a human–robot team was tasked with navigating the robot from point A to point B. The team would be faced with *in situ* events that impacted the robot’s competency, and the human supervisor would need to make autonomy-adjustment decisions in order to achieve the task goal.

**FIGURE 9:**
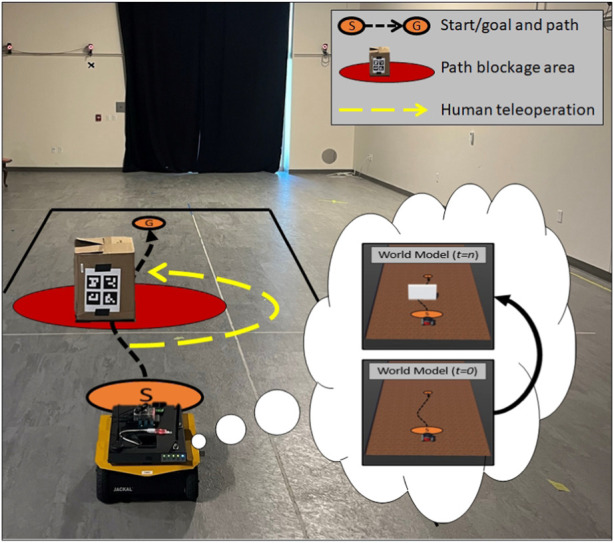
Annotated image of the ET-GOA demonstration area. The robot was tasked with navigating from the start (S) to the goal (G). A popup obstacle, shown in red, caused the ET-GOA algorithm to report low confidence. A temporary autonomy adjustment to teleoperation occurred approximately along the yellow dashed line that helped the robot around the obstacle. The thought bubble shows the robot’s world model at *t* = 0 prior to the robot learning of the obstacle and at *t* = *n* after the popup obstacle was observed and added to the world model.

The role of the human supervisor was played by the first author of this manuscript. We used the same Clearpath Jackal from our previous experiments. The robot had access (*via* ROS) to the ET-GOA capability, which enabled it to autonomously and selectively assess and report the outcome competency to the human supervisor. The scenario required the robot to autonomously plan and follow a set of waypoints through a virtually constrained space. There were two autonomy levels available to the team: 1) autonomous control, where the robot’s autonomy generated velocity commands between waypoints and 2) human control, where the supervisor teleoperated the robot using video from the front-facing camera and PlayStation controller. A single *in situ* popup obstacle blocked the robot’s ability to complete the navigation task. In other words, the robot was not capable of navigating around this obstacle and would require assistance. This could be similar to real-life situations, where falling debris or unexpected craters block the robot’s path. The popup obstacle put the team in the position of needing temporary *ad hoc* autonomy adjustment: from autonomous control to human control, as the supervisor helps the robot around the obstacle and from human control to autonomous control once the robot is back on a traversable path. Figure 9 provides an annotated image of the demonstration area. The robot is shown in the foreground, and the approximate positions of the start (S) and goal (G) are shown in orange ovals. The approximate initial path is shown as a black dashed line. A red oval depicts the area of the popup obstacle. The obstacle itself was a cardboard box with a set of AR tags that the robot used to detect the obstacle. The box was placed to block the robot when it was approximately at the second waypoint. The yellow arrow depicts the approximate location and direction of the temporary teleoperation by the supervisor to help the robot around the obstacle.

**FIGURE 10:**
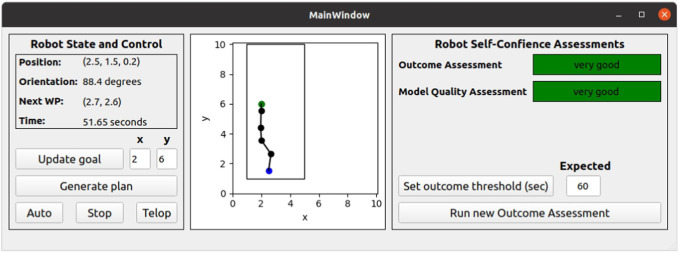
User interface for the ET-GOA demonstration. The left panel shows the robot real-time state and provides controls for planning as well as changing the autonomy level from robot control (Auto) and human control (Telop). The center panel shows the mission area, waypoint/path as black circles, the robot as the blue circle, and goal as the green circle. The right panel shows the robot’s self-assessment metrics mapped to a semantic label and includes controls to manually trigger GOA. A PlayStation controller and video interface (not shown) were used during teleoperation.

Robot supervisory control, planning, and competency assessment reports were presented to the supervisor through the user interface, as shown in Figure 10. The left panel shows the robot’s live-state telemetry data, which included position, orientation, the next waypoint, and the current mission time in seconds. There was input for choosing the goal location, generating a waypoint plan, and selecting the autonomy level (robotic autonomy or teleoperation). The center panel displayed a simple map of the mission area, the waypoints are denoted by black circles connected by a black line, the robot’s current position is denoted by a blue circle, and the goal location is denoted by a green circle. The right panel showed the robot’s real-time self-confidence for both the GOA and MQA through the ET-GOA algorithm. The right panel also had buttons to set the GOA outcome threshold and to manually query the autonomy for a competency report based on GOA. The supervisor was also given access to a PlayStation controller for *ad hoc* teleoperation.

**FIGURE 11:**
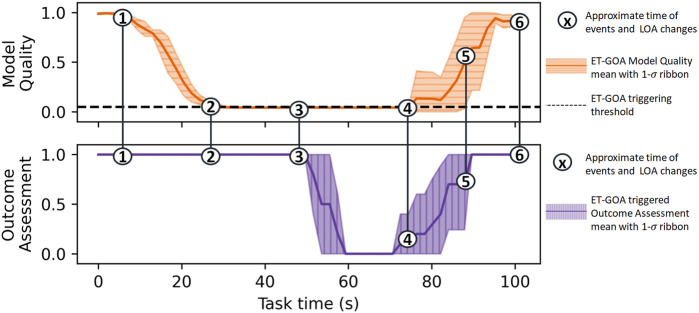
Model Quality Assessment and GOA measured across 10 live demonstrations of the ET-GOA algorithm. The robot begins with high confidence due to the world model indicating an easily navigable environment. (1) The popup obstacle appears in front of the robot; (2) the ET-GOA triggers and begins GOA; (3) GOA completes, the robot reports “very bad confidence,” and the supervisor takes control to help the robot around the obstacle; (4) the supervisor returns control to the robot, and the robot begins GOA; (5) the robot reports “very good confidence,” and the supervisor allows the robot to continue autonomous operation; and (6) the robot successfully arrives at the goal.

We executed 10 demonstration episodes. Each episode utilized the same start/end point but a unique set of waypoints generated by the *RRT* planner. We chose the ET-GOA threshold of *δ* = 0.05 because it showed a reasonable trade-off between GOA accuracy and algorithm runtime in our initial studies. The GOA outcome of interest was *total task time*, the robot’s self-confidence that it could navigate to the goal in z_*_ = 100 s. The total task time was equal to the time spent driving plus the time spent assessing. We chose to investigate this outcome to facilitate a higher-level analysis of ET-GOA in a human-in-the-loop system. Figure 11 shows the aggregate data from our demonstrations, where the numbers indicate the approximate locations of events and autonomy adjustments during the task. The robot begins the task in autonomous control mode at *t* = 0. At 1), the popup obstacle is placed in front of the robot. The robot’s camera detects the obstacle and updates the world model by emplacing a simulated obstacle approximately in front of the robot. Additionally, the robot executes a temporary stop, while the obstacle is blocking it. At 2), the ET-GOA algorithm triggers due to the increasingly unexpected state measurements: the world model’s initial predictions had the robot on a continuous trajectory, while the robot’s real state was stationary because it was blocked by the obstacle. The robot then executed GOA, which took into account the new obstacle added to the world model. At 3), the robot reported “very bad confidence” in navigating to the goal, at which point the supervisor changed the autonomy level to teleoperation control and drove the robot around the obstacle. At 4), the supervisor returned control to the robot, which then executed GOA for an updated assessment. At 5), the robot reported “very good confidence,” and the supervisor approved it to continue the remainder of the task in autonomous control mode. At 6), the robot arrived safely and successfully at the goal. This live, albeit proof-of-concept demonstration showed that the robot equipped with the Event-Triggered Generalized Outcome Assessment algorithm could monitor and report its *in situ* competency in a dynamic environment. The supervisor could, in turn, make informed autonomy-adjustment decisions based on the robot’s reported competency.

## 6 Discussion

Factorized Machine Self-Confidence is a framework and set of metrics that can enable autonomous robots to self-assess and communicate competency to human supervisors. Our results indicate that the proposed MQA metric coupled with a higher-level Generalized Outcome Assessment (GOA) may be a valuable method to improve *ad hoc* autonomy adjustments and general decision-making within a human–robot team. We found that the Model Quality Assessment can detect *in situ* misalignment between state measurements and world model predictions in both simulations and real robot operations. When there was alignment between predictions and reality, MQA tended toward 1, indicating high alignment. This was evident in the case where there were known obstacles modeled by the world model (i.e., the baseline conditions). Conversely, when there was misalignment between predictions and reality (i.e., in the other conditions), MQA tended toward 0, indicating high misalignment. We take misalignment to indicate that the robot’s predictions may not be valid, and as such, the robot may not be as competent as previously believed.

MQA provides us an indication of *when* competency may have changed but not *how* it has changed. To understand the how, we developed an ET-GOA algorithm, which uses MQA as a trigger for the robot to analyze higher-level task outcomes using GOA. Because GOA samples from a possibly complex world model, it can be computationally expensive, so we want to limit re-assessments during task execution. McGinley (2022) proposed a potential avenue to reduce computational complexity of GOA through approximation and selective sampling of the world model; however, whether these techniques translate to live platforms and dynamic operational environments is an open question.

The strong reliance on the world model paradigm presents several challenges as well. First and foremost is the existence of a world model. We provided several examples of world models in Section 3.1; however, most were a significant simplification of the “real world.” Future work toward competency assessments using world models will have to contend with trade-offs. On the one hand, simple environments with simple dynamics are easier to develop and simulate, but that simplicity may lead to inaccurate assessments. On the other hand, world models with realistic environments and dynamics could facilitate accurate robot self-assessments but may be difficult to develop and simulate. Given a world model, a second challenge is how to efficiently update it. In this work, we utilized a camera to help the robot detect and place obstacles within its world model, but this assumes that the robot is capable of detecting and understanding these obstacles in the first place. Other common approaches, such as LIDAR and RADAR, might have better detection ability but may not capture contextual and semantic information about the obstacle (for example, a concrete block and cardboard box may be the same size, but one can be driven over more easily than the other). An interesting direction could be for the supervisor to help the robot fill in any blanks caused by model simplifications or sensor limitations. This could provide a more fine-grained and collaborative way for the robot to understand the world around it and how that world impacts its competency.

We found that there are trade-offs in the choice of ET-GOA parameters a designer must make. In our experiments, a *δ* threshold close to zero provided a good trade-off between the accuracy of the triggered outcome assessments and the computational cost involved in computing those assessments. However, the choice of triggering threshold could be mission-dependent, based on how cautious the designer requires the robot to be. For example, a rover on Mars might have a higher ET-GOA threshold (more sensitive) to capture unexpected events early and often, while a food delivery robot in San Francisco might have a lower ET-GOA threshold (less sensitive) to prevent unneeded delays due to overly cautious re-assessments. Additionally, we did not vary the number of type of indicators used for ET-GOA. We believe that these may be task-dependent as well. Future work could investigate the impact of different ET-GOA indicators, as well as how to choose appropriate triggering thresholds. The choice of the indicator and threshold could even be chosen dynamically, based on factors such as mission needs or obstacle locations (Theurkauf et al., 2023).

Lastly, it is important to understand how knowledge about a robot’s competency impacts decision-making. This work is focused on improving a human supervisor’s autonomy-adjustment decisions within a human–robot team, i.e., calibrating the supervisor as to when they should rely on, or trust, the robot to operate with some degree of autonomy. Our human-in-the-loop demonstration shows that the ET-GOA algorithm can report changes in competency, which in turn may help the supervisor understand when the robot should and should not operate autonomously and thus when the robot would and would not need human assistance. Future work is needed to perform full human-subject studies in more complex deployments to validate the ET-GOA algorithm, the interaction it facilitates, and the general usability of competency assessment in realistic, live scenarios. Our work here utilized two autonomy levels: human control and robot control. However, one could imagine that monitoring changes in robot competency may help autonomy adjustment at a fine-grained level. For example, the robot reporting “fair” confidence could be a signal that the supervisor should monitor the robot’s progress more closely but not necessarily execute a control takeover. Future work could investigate how competency reporting may facilitate more fluid autonomy adjustments.

## 7 Conclusion

In this work, we investigated using self-assessed robot competency information to facilitate *ad hoc* autonomy adjustments for human–robot teams operating in dynamic environments. We presented a new MQA metric for the Factorized Machine Self-Confidence framework. We then developed an *Event-Triggered Generalized Outcome Assessment* algorithm, which used real-time computations of MQA to trigger a GOA of the robot’s confidence in achieving high-level task objectives. The GOA can be used to assist human supervisors in making *in situ*, *ad hoc* autonomy-adjustment decisions. We presented simulated and live results showing that MQA could capture unexpected perturbations to the robot state and that the ET-GOA algorithm could provide accurate online self-assessment capability for an autonomous robot. We concluded with a proof-of-concept demonstration and discussion of using ET-GOA in a human-in-the-loop system, which enabled a human supervisor to make *ad hoc* autonomy adjustments based on the robot’s reported competency. We believe that robot self-confidence can provide future human–robot teams with valuable information about the competency of the robot, which can in turn improve human decision-making and enable more effective human–robot teams.

## Data Availability

The datasets presented in this study can be found in online repositories. The names of the repository/repositories and accession number(s) can be found at: https://github.com/nickconlon/goa_robot_study.
